# Pivotal role of *Helicobacter pylori* virulence genes in pathogenicity and vaccine development

**DOI:** 10.3389/fmed.2024.1523991

**Published:** 2025-01-06

**Authors:** Ayman Elbehiry, Eman Marzouk, Adil Abalkhail, Wael Sindi, Yasir Alzahrani, Salem Alhifani, Turki Alshehri, Nuha Abdulaziz Anajirih, Turki ALMutairi, Ahmad Alsaedi, Feras Alzaben, Abdullah Alqrni, Abdelmaged Draz, Abdulaziz M. Almuzaini, Sahar N. Aljarallah, Abdulrahman Almujaidel, Akram Abu-Okail

**Affiliations:** ^1^Department of Public Health, College of Applied Medical Sciences, Qassim University, Buraydah, Saudi Arabia; ^2^Department of Population, Public and Environmental Health, General Administration of Health Services, Ministry of Defense, Riyadh, Saudi Arabia; ^3^Department of Psychiatry, King Fahad Armed Forces Hospital, Jeddah, Saudi Arabia; ^4^Department of Dental, Alhada Armed Forces Hospital, Taif, Saudi Arabia; ^5^Department of Medical Emergency Services, Faculty of Health Sciences, Umm Al-Qura University, Al-Qunfudah, Saudi Arabia; ^6^Department of Education and Training, Prince Sultan Military College of Health Sciences, Dammam, Saudi Arabia; ^7^Department of Food Service, King Fahad Armed Forces Hospital, Jeddah, Saudi Arabia; ^8^Department of Preventive Medicine, King Fahad Armed Hospital, Jeddah, Saudi Arabia; ^9^Department of Veterinary Preventive Medicine, College of Veterinary Medicine, Qassim University, Buraydah, Saudi Arabia; ^10^Department of Pharmacy Sciences, College of Pharmacy, AlMaarefa University, Riyadh, Saudi Arabia; ^11^Department of Pathology and Laboratory Diagnosis, College of Veterinary Medicine, Qassim University, Buraydah, Saudi Arabia

**Keywords:** *Helicobacter pylori*, pathogenesis, virulence factors, vaccine immunogenicity, public health

## Abstract

One of the most prevalent human infections is *Helicobacter pylori* (*H. pylori*), which affects more than half of the global population. Although *H. pylori* infections are widespread, only a minority of individuals develop severe gastroduodenal disorders. The global resistance of *H. pylori* to antibiotics has reached concerning levels, significantly impacting the effectiveness of treatment. Consequently, the development of vaccines targeting virulence factors may present a viable alternative for the treatment and prevention of *H. pylori* infections. This review aims to provide a comprehensive overview of the current understanding of *H. pylori* infection, with a particular focus on its virulence factors, pathophysiology, and vaccination strategies. This review discusses various virulence factors associated with *H. pylori*, such as cytotoxin-associated gene A (*cagA*), vacuolating cytotoxin gene (*vacA*), outer membrane proteins (*OMPs*), neutrophil-activated protein (*NAP*), urease (*ure*), and catalase. The development of vaccines based on these virulence characteristics is essential for controlling infection and ensuring long-lasting protection. Various vaccination strategies and formulations have been tested in animal models; however, their effectiveness and reproducibility in humans remain uncertain. Different types of vaccines, including vector-based vaccines, inactivated whole cells, genetically modified protein-based subunits, and multiepitope nucleic acid (DNA) vaccines, have been explored. While some vaccines have demonstrated promising results in murine models, only a limited number have been successfully tested in humans. This article provides a thorough evaluation of recent research on *H. pylori* virulence genes and vaccination methods, offering valuable insights for future strategies to address this global health challenge.

## 1 Introduction

*Helicobacter pylori* (*H. pylori*) is an ancient microbe that predates Columbus’s expeditions (1). It is a gram-negative, microaerophilic spiral bacterium first identified in the early 1980s by Australian physicians Barry Marshall and Robin Warren. In recognition of their discovery of *H. pylori* and its link to gastrointestinal disorders, including gastritis and peptic ulcers, they received the Nobel Prize in Physiology or Medicine from the Nobel Assembly at the Karolinska Institute in 2005 (2, 3). Research on *H. pylori* has advanced significantly, as scientists have strived to clarify the complexities of this infection. More than half of the global population is estimated to be chronically infected with *H. pylori*, a major public health concern because of its potential to contribute to severe health issues (4–7). The prevalence is 20 to 40% in high-income countries and 70 to 90% in low-income countries (6, 8, 9). *H. pylori* is a formidable pathogen known for causing chronic stomach infections that can last a lifetime (10, 11). Its remarkable adaptability to the acidic environment of the stomach has resulted in various host responses and pathogenic outcomes (12, 13). Initially, linked to peptic ulcers, *H. pylori* is now associated with gastritis, duodenal ulcers, stomach cancer, and multiple extragastric conditions, including neurological, ophthalmic, hematological, cardiovascular, and dermatological disorders (14–17). Millions of people worldwide suffer from these conditions, leading to substantial financial and medical burdens (18). The World Health Organization classifies *H. pylori* as a class I carcinogen, the primary cause of stomach cancer deaths globally (5, 19–21).

**Figure 1:**
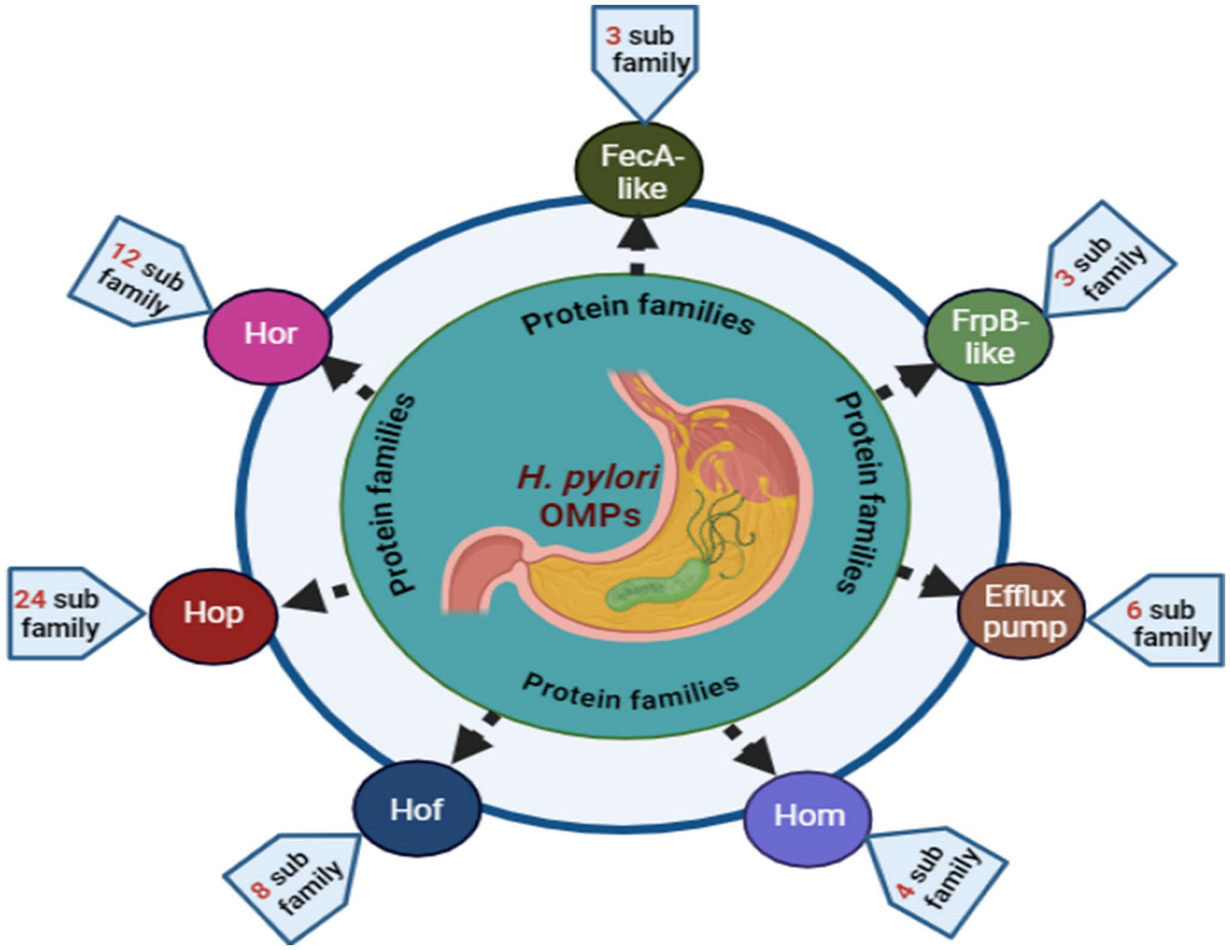
Overview of *H. pylori* OMPs. The Hop family includes 22 genes (*hopA*–*hopQ*, *hopU*, *hopZ*, *babA*, *babB*), the Hor family has 12 genes (*horA*–*horL*), the Hof family consists of 8 genes (*hofA*–*hofH*), and the Hom family contains 4 genes (*homA*–*homD*). The FecA-like and FrpB-like families each have 3 genes (*fecA-1*, *fecA-2*, *fecA-3*, and *frpB-1*, *frpB-2*, *frpB-3*, respectively). The efflux pump family comprises 6 genes: *hefA*, *hefD*, *hefG*, *flgH*, *palA*, and *lpp20*.

Virulence genes from related families, including flagella, ureases, membrane glycoproteins, and outer membrane proteins (OMPs), play a significant role in *H. pylori* pathogenicity (9, 22, 23). Four to six flagella per cell enhance mobility and gastric epithelium penetration (24). Urease secretion lowers the gastric pH and releases ammonia, creating a conducive environment for microbial colonization and potential ulceration (25). Lipopolysaccharide (LPS) improves the adherence of pathogens to the gastrointestinal mucosa, promoting infection (13, 26). OMPs are crucial for adhesion and pathogenicity, leading to inflammation (27). Sixty-four OMP gene family members, including iron-regulated OMPs and principal OMPs (Figure 1), such as *Hop*, *Hor*, *Hof*, and *Hom*, have been identified (28). Other OMPs, such as *oipA*, *sabA*, and *babA*, enhance gastric mucosa colonization (22, 29). Seo (30) reported that vaccines containing *vacA*, *cagA*, and *NAP* effectively prevented experimental infections in animal models.

Amoxicillin, clarithromycin, and metronidazole are commonly used to treat *H. pylori*-related gastric infections, often with proton pump inhibitors (29). Studies have shown that these antibiotics achieve an average eradication rate of approximately 80% (31); however, overuse may contribute to antimicrobial resistance. The resistance rates of *H. pylori* to various antibiotics were reported as follows: in the United States from 2011–2021, the rates were 42.1% for metronidazole, 31.5% for clarithromycin, 37.6% for levofloxacin, and 2.6% for amoxicillin (32). In Europe, during the period from 2008–2017, the resistance rates were 38.9% for metronidazole, 21.4% for clarithromycin, 15.8% for levofloxacin, and 0.2% for amoxicillin (33). In Africa, from 1986–2017, the resistance rates were 75.8% for metronidazole, 29.2% for clarithromycin, 17.4% for levofloxacin, and 72.6% for amoxicillin (34). Researchers are investigating innovative strategies for treating and preventing *H. pylori* infections, particularly through vaccine development. Recent studies have identified virulence determinants that may protect against infection and help eradicate bacteria in murine models (35). The evidence supports the potential use of these factors in the development of an effective human vaccine (9, 36–38). A vaccination program targeting these virulence factors could effectively manage or eliminate pathogenic strains (39, 40). Various vaccination regimens tested in animal models have shown positive results. This review explores the pathogenesis and virulence factors of *H. pylori* infection, along with the current research status and limitations of *H. pylori* vaccines.

## 2 Methodology

A comprehensive literature search was conducted to review the virulence factors, pathogenesis, and vaccines associated with *H. pylori*. The inclusion criteria included original research articles, review articles, and clinical trials focused on pathogenicity, virulence factors, and vaccine development. The key topics addressed were mechanisms of pathogenesis; immune responses; and specific virulence factors, such as *OMPs*, *cagA*, *vacA*, *NAP*, *ure*, and catalase. Only English-language publications from 1989–2024 were included, whereas nonresearched materials, non-English publications, and duplicate studies were excluded. Searches were performed in databases such as PubMed, Web of Science, Scopus, and Google Scholar, using terms such as “*H. pylori*,” “pathophysiology,” “antimicrobial resistance,” “virulence factors,” “vector vaccine,” “subunit vaccine,” and “DNA vaccine.”

## 3 *Helicobacter pylori* pathogenesis and the immune response

**Figure 2:**
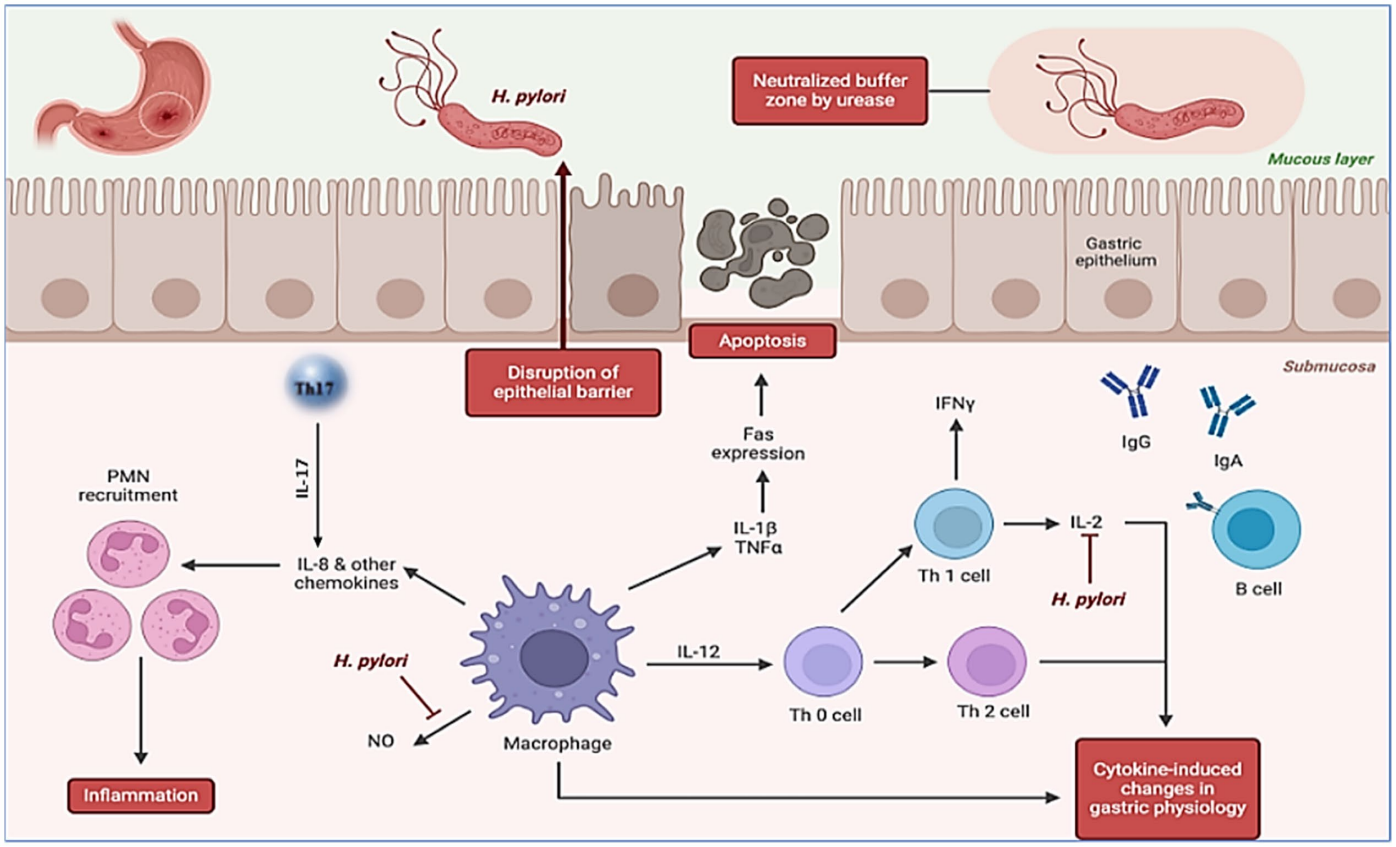
The pathogenesis of *H. pylori* is illustrated in the accompanying figure, which has been carefully edited and designed utilizing dynamic elements from BioRender. Created with BioRender.com

The interaction of the host immune system with bacterial components leads to an immunological response to *H. pylori* infection (41, 42). This triggers a complex local inflammatory response in the stomach, which is typical of *H. pylori* infections (7, 43, 44). During the innate immune response, *H. pylori* causes a persistent inflammatory reaction in the gastric mucosa (Figure 2). The relationship between LPS and peptidoglycan in the *H. pylori* cell wall is essential for this response, which is marked by the infiltration of immune cells such as neutrophils, macrophages, and lymphocytes and the release of proinflammatory cytokines such as IL-1β, IL-6, IL-8, and TNF-α (45, 46). Moreover, IL-17 plays a crucial role in the immune response to *Helicobacter* infections in both humans and mice (47, 48). In humans, IL-17 induces the secretion of IL-8 by activating the ERK 1/2 MAP kinase pathway, and the released IL-8 attracts neutrophils, promoting inflammation (49). IL-17 has two main roles: T regulatory cells modulate inflammation to support bacterial survival, and vaccination generates *Helicobacter*-specific memory T helper cells, increasing IL-17-mediated inflammation and assisting in bacterial clearance (49). The enhanced proinflammatory effects of IL-17 by CD4^+^ cells can significantly help eradicate bacteria in murine models (50, 51). Compared with unvaccinated mice, vaccinated mice exhibit higher IL-17 mRNA levels in their stomachs (52). Innate immune responses are activated by pattern recognition receptors, such as Toll-like receptors, which detect bacterial components such as LPS and peptidoglycan, triggering inflammatory reactions (53). The adaptive immune response follows, with CD4^+^ T helper (Th) cells stimulating a Th1 response that secretes interferon-gamma (IFN-γ). B lymphocytes produce antibodies against *H. pylori*, and regulatory T cells help modulate infection (54–56).

Bacteria use various immune evasion strategies, including urease synthesis and molecular mimicry, to avoid detection by T and B cells (57, 58). Prolonged *H. pylori* infection causes chronic inflammation and damage to the gastric mucosa. Increased turnover of gastric epithelial cells contributes to ulceration and mucosal injury (59). This compromises mucosal barrier integrity due to tight junction disruption. *H. pylori* penetrates the mucosal layers, facilitating rapid spread. The continuous release of inflammatory mediators and genomic instability foster an environment conducive to cancer development (60). *H. pylori* disrupts local immune responses, which may lead to malignancies in the gastric epithelium (61).

## 4 Certain virulence factors associated with *Helicobacter pylori* infection

Certain virulence antigens are associated with the severity of symptoms and clinical outcomes in *H. pylori* infections. Antigens of *H. pylori*, such as *cagA*, *vacA*, *NAP*, *OMPs* (e.g., *babA*, *sapA*, *oipA*), urease, catalase, and Hsp60, are also considered potential candidates for vaccines. These antigens trigger both humoral and cellular immune responses during infection. This section provides an overview of their roles in invasion, survival, colonization, and inflammation in the gastric mucosa as well as their role in improving vaccine development.

### 4.1 *cagA* and *vacA* genes

The virulence factors *cagA* and *vacA* of *H. pylori* are crucial for cytotoxin production (62). These genes are part of the type IV secretion system (T4SS), which is vital for bacterial pathogenicity (63). T4SSs are complex structures that penetrate bacterial cell walls, aiding survival and protein or DNA translocation (64). Approximately 60–70% of *H. pylori* strains express the *cagA* protein, which produces a specific cytotoxin (65). Phosphorylation of tyrosine motifs in *cagA* allows its translocation into gastric epithelial cells via T4SSs. Research by Yamaoka et al. (61), Selbach et al. (66), and Stein et al. (67) indicates that variations in these motifs are linked to gastric degeneration and increased gastric cancer risk. Phosphorylated *cagA* triggers pathological responses in host cells, enhancing motility, actin polymerization, and cell stretching and disrupting physiological signals (68). *cagA* influences Th17 cell differentiation by interacting with the STAT3 protein, which is crucial for T and B lymphocyte development. It also interacts with NF-κB, a key regulator of innate immune responses (69). *cagA* activates Th1 and Th17 cells to eliminate *H. pylori* and promotes proinflammatory cytokine expression in gastric epithelial cells (70). *H. pylori* strains with *cagA* enhance IL-8 secretion (71). The high immunogenicity of *cagA* is linked to increased gastric inflammation (72), which negatively affects *H. pylori* survival (73). Furthermore, *cagA* promotes a Th1-polarized immune response that aids infection clearance (74). This dual role indicates that *H. pylori* must regulate *cagA* expression during gastric colonization. The *cagA* gene is vital for vaccine development beyond its pathogenic properties. Paydarnia et al. (75) studied the effects of mixed immunization with *H. pylori* LPS and recombinant *cagA* on immune responses in a murine model. The recombinant *cagA* protein, given with a cytosine phosphoguanine adjuvant, maintained its antigenic properties and triggered strong Th1-biased immune responses throughout the experiment. These findings suggest that *cagA* may be key to an effective vaccine for *H. pylori* infection.

Most *H. pylori* isolates contain the *vacA* gene, which targets epithelial and immune cells in the digestive tract (76, 77). Like the *cagA* gene, *vacA* is unique to type I *H. pylori*. The *vacA* protein not only facilitates intracellular vacuole formation but also has toxic effects on various cells (78, 79) and survives the acidity of the stomach via multiple exit routes (79). Although the exact mechanisms by which *vacA* induces autophagy are not fully understood, it has been shown that the autophagy triggered by *vacA* is dependent on its interaction with low-density lipoprotein receptor-related protein 1 (80). The *vacA* protein affects apoptotic signaling in host cells, limiting apoptosis. Its influence can be proapoptotic or antiapoptotic, depending on the cell type and environment. The *vacA* toxin also alters host cell morphology and function by inducing vacuole formation (78). Additionally, toxins hinder T cells and other immune cells, impacting the overall immune response (81, 82). In most *H. pylori*-infected patients, anti-*vacA* antibodies are found in their blood and gastric juice (83). The growth of CD4^+^ lymphocytes from the gastric epithelium is antigen dependent when *vacA* is present (84, 85). While *vacA*-induced T and B-cell responses are detectable, they do not eliminate *H. pylori* infection. However, these immune responses indicate that *vacA* is immunogenic in humans and may be a candidate for vaccines. Therefore, the *vacA* gene also plays a role as a protective factor against *H. pylori* infection (86). Moyat and Velin (85) reported that a *vacA*-based vaccine showed significant protective effects on infected mice that received therapeutic intragastric immunization with a nontoxic recombinant version of *vacA* and the LT mutant LTK63. For the majority of vaccinated individuals, it effectively eliminates *H. pylori* infection and reduces the risk of reinfection (87). Preventive vaccination in animal models has also shown promise, with recombinant *vacA* and mucosal adjuvants providing protection (88).

The interaction between the *cagA* and *vacA* proteins significantly contributes to *H. pylori*-associated gastric cancer. Abdullah et al. (89) reported that the absence of *vacA* allows the host immune system to degrade *cagA*, preventing its accumulation in gastric epithelial cells. *H. pylori* infection increases the risk of gastric cancer, posing a major public health challenge. There is a strong link between cancer progression and the growth of other gastric malignancies, driven by inflammation, genotoxic factors, and genomic instability (90). This relationship is influenced by host genetics, environmental conditions, and *H. pylori* virulence genes such as *oipA*, *vacA*, and *cagA* (62). Understanding these pathways is crucial for developing effective treatments and preventing future infections. Recent advancements are improving our knowledge of *H. pylori*-related diseases and aiding the development of innovative therapies, including potential vaccines.

### 4.2 Neutrophil-activating protein

In all strains of *H. pylori*, a 150 kDa multimeric protein, referred to as neutrophil-activating protein (*HP-NAP*), has been identified (5, 91, 92). Research indicates that *HP-NAP* enhances the penetration and generation of oxygen radicals and the adhesion of neutrophils and monocytes to gastric endothelial cells while also increasing their motility (93). This activity contributes to long-term inflammatory conditions in the gastrointestinal epithelium. *HP-NAP* activation leads to increased interleukin-12 (IL-12) production, triggering a T helper 1 (Th1) immune response (94). Immunodominant antigens associated with the *H. pylori* G27 strain were identified through two-dimensional gel electrophoresis in a patient suffering from various gastric diseases, with this protein being significantly recognized in the serum of infected individuals (95). Furthermore, animals immunized with *HP-NAP* have demonstrated immunity against subsequent infections, indicating that this virulent gene may serve as a promising candidate for vaccine development. Owing to its high antigenicity, *HP-NAP* is frequently incorporated into vaccines aimed at preventing *H. pylori* infection (94). In addition to its application in vaccines, *HP-NAP* may also hold potential as an immunotherapeutic agent in cancer treatment, as its immunomodulatory properties enable dendritic cells to promote Th1 responses and enhance the immune responses of recipients (94).

### 4.3 Outer membrane proteins

OMPs of *H. pylori* are essential for physiological processes, assisting in material transport and host interactions (96, 97). They are promising targets for vaccines and medications (9, 98, 99). *H. pylori* has diverse OMPs, such as lipoproteins, porins, and adhesins, which are vital for survival and pathogenicity (27). OMP expression varies among strains, contributing to pathogenicity through adherence, invasion, and immune evasion (26, 96). Genome sequencing revealed that approximately 4% of *H. pylori* genetic material encodes OMPs, which are categorized into five gene families: *Hop*, *Hor*, *Hom*, *Hof*, and iron-regulated proteins (27, 100, 101). This section summarizes recent advancements in understanding well-characterized OMPs.

#### 4.3.1 Blood group antigen-binding adhesin

Blood group antigen-binding adhesin (*babA*) is part of the *Helicobacter* Hop family and plays a crucial role in *H. pylori* adhesion (102, 103). Currently, three genetic variants of the *bab* gene have been identified: *babA1*, *babA2*, and *babB* (104). Three genetic variants of the *bab* gene exist: *babA1*, *babA2*, and *babB* (27, 62, 102). The *babA2* gene encodes a significant adhesin that binds to Lewis b (Le-b) blood group antigens, aiding colonization and bacterial density. Strains with *babA1* do not express *babA*, whereas those with *babA2* can be poor or significant producers of *babA*, affecting adhesion to Le-b antigens. Genomic analysis revealed that *babA* and *babB* are unrelated, with their expression levels varying geographically (62). The prevalence of the *babA2* gene ranges from 44.0% in Portugal to 79.7% in Iran, with only 9.8% of Western strains lacking *babA* (105). Studies have linked *H. pylori* to diseases such as gastric cancer, resulting in increased *babA* expression in affected patients, suggesting its role in disease severity (27, 106, 107). These observations suggest that *babA* may play a role in the severity of disease outcomes associated with *H. pylori* infection (108). Additionally, the T4SS may facilitate *cagA* penetration through the gastrointestinal epithelium via the interaction between *babA* and Le-b (109). *babA* is a crucial factor in *H. pylori* infections and could be further investigated as a potential preventative treatment and vaccine candidate (102, 110, 111). Bai et al. (112) successfully isolated recombinant *babA2* from the serum of patients infected with *babA2*-positive *H. pylori*, as well as from BALB/c mice infected with recombinant *babA*. This discovery suggests that *babA2* could be a promising vaccine antigen because of its immunogenic properties.

#### 4.3.2 Sialic acid-binding adhesin

The sialic acid-binding adhesin (*sabA*) gene in *H. pylori* is the second most prevalent OMP (27) and has two alleles: *sabA* (*HopP* or *OMP17*) and *sabB* (*HopO* or *OMP16*), both of which are part of the Hop protein family (113). *sabA*, which is smaller than *babA* at approximately 70 kDa (102), detects and binds to sialylated glycans, particularly sialyl Le-x antigens (114). As a sialic acid-binding adhesin, *sabA* interacts with host cell receptors. *H. pylori* strains often carry both *sabA* and *sabB*, indicating preferential expression of *sabA* during colonization (115, 116). *sabA* is increasingly recognized as crucial in gastrointestinal disease pathogenesis (117). *H. pylori* infection likely begins with *babA* binding to fucosylated antigens related to the ABO blood group and the Le-b antigen (102). Furthermore, sialyl-Le-X expression increases during the host inflammatory response, enhancing *H. pylori* adhesion to the gastric mucosa alongside sabA activity (102, 118, 119). Research in developed and developing nations has linked *sabA* generation to severe gastrointestinal diseases, gastric atrophy, and gastric cancer (120). Further investigations into *sabA* are urgently needed, particularly in developing countries. *sabA* also mimics selectin, activating neutrophils and producing reactive oxygen species (ROS), which prolong inflammation (121).

*H. pylori* colonizes the gastric mucosa more readily in the presence of gamma-glutamyl transpeptidase (GGT), which induces programmed cell death in gastric epithelial cells (5, 122, 123). GGT also impairs dendritic cell development and T-cell-mediated immunity, enhancing resistance to infection. Additionally, *H. pylori* transports GGT in outer membrane vesicles, increasing hydrogen peroxide and interleukin-8 (IL-8) production in gastric epithelial cells (26, 93, 124). Multiepitope vaccines contain antigenic epitopes from the virulence factors of *H. pylori*, such as the *sabA* and *babA* genes (102). Urrutia-Baca et al. (111) developed an oral vaccine with 11 epitopes linked to pathogenicity and colonization, including *babA* and *sabA*. Modeling studies suggest that this vaccine candidate will show antigenicity, nonallergenicity, and solubility, with an appropriate molecular weight. A study by AlEraky et al. (125) identified antigenic peptides from *H. pylori* for vaccine development via an *in silico* proteomic method. Four peptides—*cagA1*, *cagA2*, *vacA*, and *sabA*—were further investigated through reverse vaccinology. After immunization with these peptides and Freund’s adjuvant, BALB/C mice were orally challenged with *H. pylori*. *sabA*-vaccinated mice presented significantly higher IgG and IL-4 levels than did the adjuvant-only group. Histopathological evaluations revealed a protective immune response in the vaccinated groups, particularly with the *sabA* antigen. However, further *in vitro* and *in vivo* studies are needed to assess its efficacy before its use in humans (111).

#### 4.3.3 Outer inflammatory protein A

Gastric cancer development is linked to the outer membrane proteins of *H. pylori*, particularly outer inflammatory protein A (*oipA*), which includes *HopB*, *HopQ*, and *HopH* (27, 126, 127). *oipA*, encoded by the *HopH* gene, is associated with gastric mucosa inflammation. Compared with *oipA*-negative strains, *H. pylori* strains that are *oipA* positive provoke a stronger inflammatory response (124), which is correlated with a greater risk of gastric ulcers and cancer (128). *oipA* is more prevalent in gastric biopsies from cancer patients than in those from uncomplicated gastroenteritis patients (124). It also induces B-cell lymphoma-2 (Bcl-2) family proteins, contributing to apoptosis (93, 129), and upregulates inflammatory cytokines such as IL-6, IL-8, and IL-1. Additionally, Sukri et al. (130) reported that gastric cancer influences T cell, B cell, and dendritic cell development and IL-10 release, increasing cancer risk. A meta-analysis by Liu et al. (131) revealed a strong link between the presence of *oipA* and the risk of peptic ulcer disease, especially in Western countries. Several studies have evaluated *oipA* as a potential vaccine against *H. pylori* infection (3, 132). Soudi et al. (133) tested recombinant *oipA* with propolis as an adjuvant in a mouse model at doses of 10 μg/mL and 40 μg/mL. They reported that *oipA* effectively induces IFN-γ production and enhances the cellular immune response, with propolis acting as a beneficial adjuvant. Another study reported the production of anti-*oipA* IgA antibodies in C57BL/6 mice (134).

### 4.4 Urease

*H. pylori* urease (*ure*), comprising 10 to 15% of a bacterium’s total protein, consists of 12 heterodimers formed by the *ureA* and *ureB* enzymes (135). This catalytic enzyme hydrolyzes urea into carbon dioxide and ammonia, which neutralizes excess stomach acid, inhibits neutrophil activity (136–138), promotes the production of toxic ammonia-derived compounds (139), and disrupts stomach epithelial cell interactions (140), fostering bacterial colonization. The peroxynitrite anion can harm bacteria, but carbon dioxide mitigates this effect, aiding colonization (93, 141). The unique surface structure of the urease complex allows *H. pylori* to interact with host immune elements, ensuring its indefinite colonization. Inhibiting urease function prevents *H. pylori* from thriving, providing therapeutic and preventive strategies against infection (135). In numerous studies, urease has been identified as a potential antigen candidate for vaccine production. In a laboratory model of *H. pylori* infection, Nasr-Esfahani et al. (142) demonstrated that the recombinant plasmid pcDNA3.1 (+)-*ureA* could induce an immune response in murine models. Furthermore, the vaccination of mice with a recombinant *ureB* vaccine, which incorporates plant polysaccharides as adjuvants, was found to confer immunity against *H. pylori* infection. This protective effect may be attributed to the enhancement of Th1/Th17 CD4^+^ T-cell activation and the promotion of gastrointestinal-specific secretory immunoglobulin A (143). Most vaccines that have advanced to the clinical trial phase include the urease antigen (37, 135, 144–146).

### 4.5 Catalase

Catalase is a key enzyme that protects bacteria from hydrogen peroxide. In *H. pylori*, this tetrameric protein makes up approximately 1% of the total protein and has an isoelectric point of 9.0–9.3 (135, 147). It shields *H. pylori* from host-produced reactive oxygen species and helps bacteria evade macrophages (148, 149). Its role in various pathological processes contributes to inflammation, apoptosis, and tumor formation, with mutagenesis occurring in the cytoplasm, periplasm, and occasionally on the surface (150). *H. pylori* catalase is one of its most highly expressed proteins and shows greater resistance to cyanide and amino triazole suppression than do catalases from other bacteria (124). Recent studies have provided detailed characterizations of immunodominant Th1 epitopes associated with catalase (151). Through the production of IFN-γ, seven novel catalase epitopes have been identified as potent inducers of a robust Th1 immune response (152). The LHUC vaccine is a multivalent epitope vaccine that incorporates the adjuvant heat-labile enterotoxin B subunit, along with five B-cell epitopes and three Th-cell epitopes (*HpaA*, *ureB*, and catalase), designed to create an effective multivalent epitope vaccine against *H. pylori* (135). Following the administration of the LHUC vaccine to mice, serum analysis revealed the presence of antibodies specific to the antigen, accompanied by a significant increase in the production of IFN-γ, IL-4, and IL-17 by lymphocytes. Studies have demonstrated that LHUC is highly effective in preventing *H. pylori* infections in murine models (153).

## 5 *Helicobacter pylori* vaccine types

The increase in antibiotic resistance underscores the need to explore virulence factors as alternative vaccine targets for *H. pylori* infections. Understanding these factors is crucial for advancing vaccine development and effective therapies. While various vaccine types, such as vector-based, whole-cell, and subunit vaccines, have shown efficacy in animal models, few have reached human clinical trials. This section reviews recent *H. pylori* vaccine developments and the role of virulence genes in potential vaccine formulations.

### 5.1 Inactivated whole-cell vaccines

Inactivated whole-cell vaccines for *H. pylori* are created by disrupting the bacteria with ultrasonic waves and inactivating them with formalin (29). These vaccines reduce *H. pylori* proliferation and elicit strong immune responses in the gastric mucosa. Kotloff et al. (154) suggested that the administration of an oral *H. pylori* whole-cell vaccine can effectively stimulate both mucosal and systemic immune responses in humans. Murine experiments performed with whole-cell vaccines demonstrated that these vaccines can elicit a dose-dependent response, including the production of cross-reactive IgG, against *H. pylori*. The high-dose Hp 26695 whole-cell vaccine group presented reduced bacterial colonization in challenge experiments with SS1 (155). Oral vaccination is preferred for its ease of use and high adherence rates (156–159). However, it requires higher antigen dosages than intramuscular injections do, which may cause immunological tolerance. Researchers often use lower antigen doses with mucosal adjuvants to improve efficacy (160, 161), especially against *H. pylori* (29).

Aluminum adjuvants are essential in vaccine formulations, enhancing systemic immunity and promoting a Th2-type response (162, 163). Cholera toxin is a potent mucosal adjuvant (164) that activates a Th2 response (165) but poses toxicity risks (35). Cholera toxin B is a safer alternative (157, 166). Oral immunization with cholera toxin and bacterial antigens increased antibody levels in a germ-free mouse model of *H. felis* infection (167), and Lee et al. (168) reported that this combination was more effective than the *H. felis* antigen or adjuvant alone. Holmgren’s et al. (164) developed a novel adjuvant with mutant cholera toxins for *H. pylori* infections. A formalin-inactivated whole-cell *Helicobacter* vaccine increased serum IgG, mucosal IgA, IFN-γ, and IL-17 levels while reducing *H. pylori* colonization (169, 170). Improving vaccine delivery methods is vital for effective mucosal vaccination (171). Techniques such as liposomes, viral vectors, and attenuated bacterial vectors offer unique benefits. However, no commercially available vaccine exists for inactivated whole-cell *H. pylori*, and enhancing the immune response while ensuring safe delivery is challenging.

### 5.2 Genetically modified protein-based subunit vaccines

Antigenic subunit vaccines use purified pathogen components to trigger a strong immune response (172). They contain only antigenic elements, enhancing safety by removing live components (173). These vaccines are suitable for individuals with compromised immune systems (174) and have a complex manufacturing process that often requires booster doses, adjuvants, and significant time to determine optimal antigen combinations (175). The development of a subunit vaccine for *H. pylori* is particularly challenging and costly (29). Genetic engineering improves the purification and large-scale production of specific antigens, enhancing vaccine efficacy (176, 177). Key antigens for an *H. pylori* vaccine include urease, catalase, *cagA*, *babA*, *vacA*, and *fliD* (110, 178, 179). Urease was one of the first antigens recognized as beneficial for vaccine development (145, 180). Urease subunits A and B (*ureA* and *ureB*) are vital for colonization and are therapeutic targets (110, 181). While *ureB* is well studied as a vaccine candidate (145, 182, 183), research on *ureA* is limited. *ureA* activates urease via interactions with HSP60, which is crucial for protein balance (184). Murine studies suggest that *ureA*-specific CD4^+^ T cells provide protective immunity (185), and oral immunization with *Bacillus subtilis* spores expressing *ureA* has shown protective effects in trials (186).

Michetti et al. (180) demonstrated that an oral *H. pylori* urease vaccine, along with *Escherichia coli* heat-labile enterotoxin as an adjuvant, elevated anti-urease serum immunoglobulin A titers in twenty-six *H. pylori*-infected participants. Zhong et al. (187) developed a recombinant fusion protein, *ureA*-*ureB*-*NAP*, as a preventive vaccine, showing improved protection in mice compared with a bacterial lysate vaccine. Skakic et al. (179) examined protein nanocapsules with the A subunit of *H. pylori*-*ureA* and reported that TiterMax with SC/MS nanocapsules significantly reduced gastric *H. pylori* infections in murine models, indicating effective immune response stimulation. The role of *H. pylori oipA* in promoting the proinflammatory cytokine IL-8 and its effect on inflammation has been studied (188). While *oipA* is vital for host protection against *H. pylori* (134), selecting an effective adjuvant is crucial for a strong immune response. Many vaccines still use oil emulsions or aluminum salts, but research into alternatives continues due to the adverse effects of oil-based adjuvants (189). Natural adjuvants such as propolis have shown promise in animal models (190) and may enhance vaccination strategies. A 2021 study revealed that propolis combined with recombinant *oipA* increased IFN-γ production and strengthened the immune response (133).

### 5.3 *Helicobacter pylori*-NAP in vaccination

The *H. pylori*-*NAP* gene is a key virulence factor and a potential target for gastrointestinal disorder treatments (5, 191). Its immunological properties also suggest potential for cancer treatment and *H. pylori* infections (94). Guo et al. (192) studied multivalent epitope-based vaccines in Mongolian gerbils in 2017 and 2019 (193). The first vaccine combines *H. pylori-NAP* with various antigens, while the second includes epitopes from *cagA*, *vacA*, and urease, both of which effectively reduce bacterial colonization and gastritis. Liu et al. (194) developed a multivalent vaccine with *H. pylori*-*NAP*, a mucosal adjuvant, and *ureA* and *ureB*, which stimulate mucosal IgA and specific humoral immune responses. Chen et al. (195) reported that cyclic guanosine monophosphate-adenosine monophosphate provided protection against *H. pylori* at lower dosages via intranasal immunization. The immunogenic properties of *H. pylori*-NAP are promising for vaccine development (94), but mixed results indicate that more research is needed to understand *H. pylori* evasion of host antibody responses.

### 5.4 *cagA* antigen as a vaccine candidate

*cagA* is a potential antigen that triggers immune responses in clinical trials (196, 197). Like other *H. pylori* proteins, such as *ureA*, *babA*, *sabA*, and *oipA*, *cagA* is an effective vaccine antigen that inhibits *H. pylori* proliferation when combined with suitable adjuvants (198, 199). In animal models, vaccination with recombinant antigens such as *cagA*, *vacA*, and *NAP* has shown protective effects, enhancing T-cell memory and cell-mediated immune responses. A study of healthy volunteers vaccinated with a *cagA*-positive strain revealed limited protection after exposure (197). Paydarnia et al. (75) revealed that recombinant *cagA* and *H. pylori* LPS stimulate host immunity in murine models. The recombinant *cagA* protein with the CpG adjuvant maintained its antigenicity and induced strong Th1-biased immune responses. Other antigens, such as *HpaA*, *FlaA*, *SOD*, and *Hsp*, may also enhance the immune response to *H. pylori* infection.

### 5.5 Multiepitope DNA vaccines

**Figure 3:**
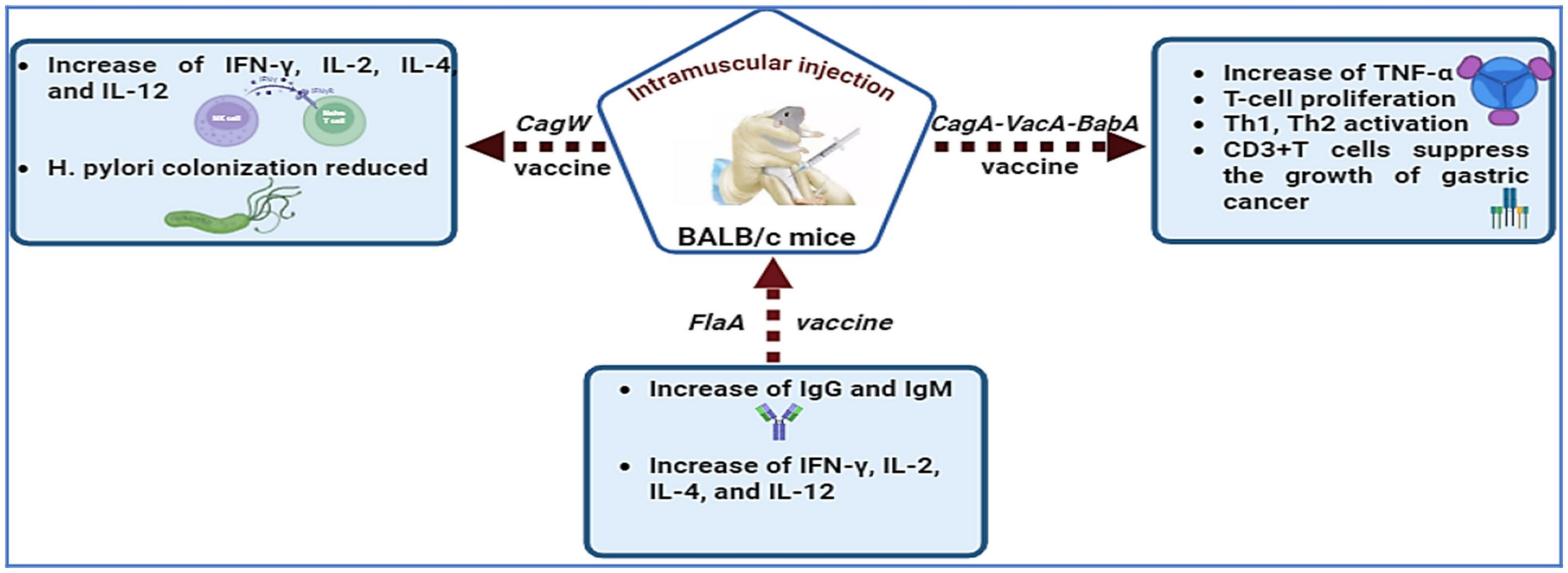
*H. pylori* DNA vaccines, including *cagW*, *cagA-vacA-babA*, and *flaA*, have been developed to enhance immunological responses.

Researchers are exploring ways to increase DNA vaccine efficacy through adjuvants, cytokines, chemokines, CpG incorporation, and electroporation (200). Nonmethylated CpG motifs in plasmid backbones increase vaccination success by stimulating immune responses in B cells and natural killer cells (201). The protective antigen of *H. pylori* is encoded by cDNA in an expression vector that is absorbed by host cells, activating the immune response (29). Kumari et al. (202) stated that the absence of *cagW* disrupts pilus formation, preventing *cagA* from entering the bacterial membrane. *babA* is crucial for *H. pylori* adherence to gastric epithelial cells and may worsen gastritis by promoting *cagA* translation (107), making it a promising vaccination target (29, 203, 204). Xue et al. (205) developed plasmid vaccines targeting *cagA*, *vacA*, and *babA* in albino mice, which showed potential anticancer properties for gastric cancer immunotherapy. Figure 3 presents recent advancements in *H. pylori* DNA vaccines, including *cagW*, *cagA*-*vacA*-*babA* (205), and *flaA* (206). However, challenges such as degradation by deoxyribonucleases, delivery issues, and limited immune responses in some primates hinder DNA vaccine efficacy. Future advancements are expected to improve clinical trial prospects for these vaccines.

### 5.6 Vector (carrier) vaccines

**Figure 4:**
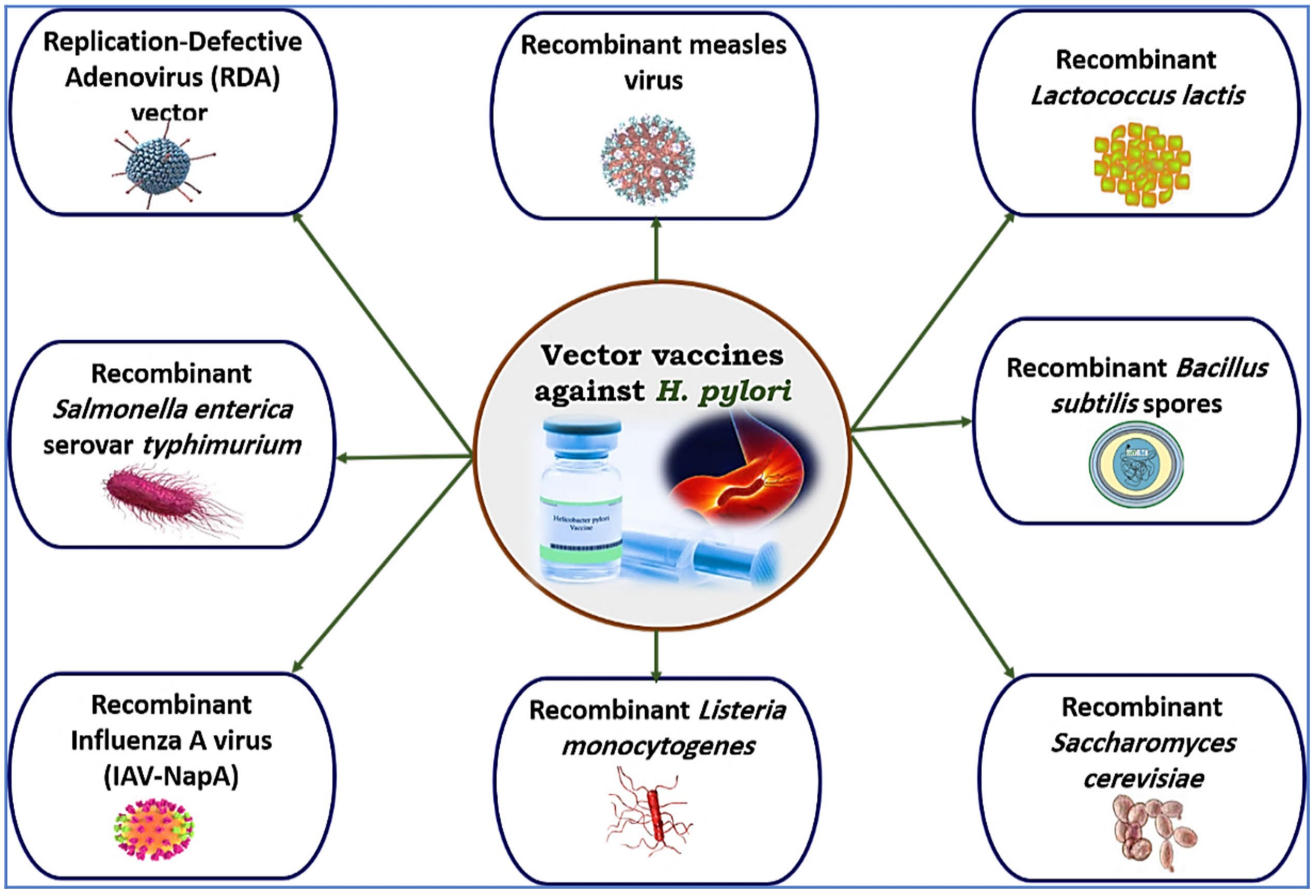
Some vector (carrier) vaccines used in murine models in trials to protect against *H. pylori.*

Both viruses and bacteria can harm human health. Modifying virulence-associated genes while preserving infectious properties at the mucosal barrier is one strategy to reduce pathogen effects. *H. pylori*-derived immunogens can stimulate an immune response when delivered to antigen-presenting cells. Vector-based vaccines can mimic natural infections and sustain immune activation, making live vectors a promising alternative to mucosal adjuvants in recombinant subunit vaccines (169). This section reviews vector vaccines for preventing *H. pylori* infection (see Figure 4).

Intramuscular administration of a replication-defective adenovirus vector can reduce *H. felis* infection spread (207); however, uncertainties remain about the immune response from poliovirus replicons with the *ureB* component (208). Research shows that intranasal or oral *Salmonella enterica* serovar Typhimurium-vectored vaccines effectively prevent *H. pylori* colonization (209–211). This method allows for needle-free vaccination and promotes the use of recombinant antigens and DNA vaccine vectors (212, 213). While effective in animal models (214, 215), these vaccines have shown limited efficacy in humans. There is a need for *Salmonella* strains that produce protective antigens for comprehensive host immunization. Ghasemi et al. (216) reported that a vaccine with *hpaA*, *H. pylori*-*NAP*, *ureA*, and *ureB* conferred protection against *H. pylori* SS1 in 70% of tested mice.

Nie et al. (217) developed the intranasal influenza A virus (IAV) vector vaccine *IAV*-*NapA*, which uses two live attenuated influenza viruses to express the *H. pylori NapA* A subunit. The strains WSN-NapA and PR8-NapA exhibited significant attenuation and strong immunogenicity in mice, inducing robust Th1 and Th17 immune responses, as well as antigen-specific humoral and mucosal responses. The vaccine effectively reduced *H. pylori* colonization and inflammation, suggesting its potential as a dual-purpose vaccine for influenza and *H. pylori*. Lactic acid bacteria serve as effective carriers for oral vaccines (218, 219), enhancing immunization due to their durability and resistance to gastric acid (220). Furthermore, the recombinant measles virus (MV) vaccine expressing the *H. pylori HspA* antigen has demonstrated significant cancer efficacy and strong immunogenicity (221), making MV a promising platform for vaccine development.

Vaccines using *Lactococcus lactis* (*L. lactis*) increase mucosal immunogenicity (222), and modified strains can trigger immune responses at both the mucosal and systemic levels (223). Zhang et al. (224) studied a recombinant *L. lactis* LL-plSAM-WAE vaccine in BALB/c mice, which expressed the SAM-WAE antigen. This vaccine induced antibodies against *H. pylori* virulence factors and activated T cells, indicating strong potential for *H. pylori* vaccine development in clinical trials. *Bacillus subtilis* (*B. subtilis*) spores are effective vectors for mucosal vaccination (186, 225, 226). Oral or nasal administration enhances mucosal immunity, particularly Th1 responses, and increases secretory immunoglobulin A (sIgA) production (227). These spores can germinate under suitable conditions and endure extreme environments, including gastric secretions (228). Thus, animals and humans are likely exposed to low levels of *Bacillus* (229). In a study by Katsande et al. (186), mice were given genetically modified *B. subtilis* spores expressing the *H. pylori* antigens *ureA* and *ureB*, leading to specific mucosal responses, increased fecal sIgA levels, and increased antibody production.

*Saccharomyces cerevisiae* (*S. cerevisiae*) is a promising candidate for immune studies against various pathogens (230). Cen et al. (231) used *S. cerevisiae* to express recombinant *ureB* and *vacA*, creating an oral vaccine that significantly reduced *H. pylori* infection in mice. Attenuated *Listeria monocytogenes* (*L. monocytogenes*) is an effective vector for enhancing antibody production against *H. pylori* (232). It stimulates immune responses, especially from CD4^+^ and CD8^+^ T lymphocytes (233), and is widely used as a vaccine carrier in immunotherapy for tumors and infectious diseases (234). The hly gene promoter (P_hIy_), encoding listeriolysin O, is often used for developing vaccine strains for foreign antigen production (235), but Ding et al. (236) reported that it is inadequate for optimal antigen expression and strong immune responses. Live attenuated bacteria must survive acidic environments such as macrophage phagosomes and the gastric cancer microenvironment (237). A key limitation is the lack of a well-characterized promoter array for regulating foreign antigen transcription in *L. monocytogenes*. Ma et al. (238) identified 21 potential promoters from *L. monocytogenes* cultured at pH 7.4 and 5.5, with seven intrinsic promoters outperforming P_help_ and five constitutive promoters showing high activity in the production of *ureB*, an antigen against *H. pylori*.

## 6 Challenges in developing *Helicobacter pylori* vaccines and moving from animal models to clinical trials

The effectiveness and immunogenicity of human vaccines differ from those of animal vaccines, creating translational challenges. No *H. pylori* vaccine candidates have advanced to human clinical trials, with many discontinued (135). Factors affecting *H. pylori* include immune evasion, genetic variations, its intracellular presence, and limited funding. T-regulatory cells are essential for a nonharmful relationship with *H. pylori*, but their responses may inhibit Th1 and Th17 functions (239), potentially promoting *H. pylori* proliferation. Genetic variations account for approximately 30% of the heterogeneity among *H. pylori* isolates (240), leading to significant genotype variability among individuals and within the same patient. The intracellular presence of *H. pylori* is also a critical factor to consider (241). *H. pylori* is commonly found in the stomach layers, epithelium, and immune cells of patients with gastric disorders (242) and often outnumbers and prolongs the lifespan of immune cells (243). Mouse models are inadequate for studying *H. pylori* (244), and Amalia et al. (245) highlighted the need for better models, as murine immune responses do not reflect human responses (240). Domestic monkeys share similarities with humans regarding *H. pylori*, suggesting that larger mammals may be more suitable for vaccine studies (246). Despite the prevalence of *H. pylori*, major biopharmaceutical companies are reluctant to develop vaccines because of challenges in assessing immune protection, genetic diversity, and host responses, complicating manufacturing.

## 7 Ethical challenges in vaccine trials for vulnerable populations

Research involving vulnerable populations requires a careful approach to prevent exploitation, especially in vaccine trials. This is critical in developing countries, where socioeconomic disadvantages can lead to coercion. Past ethical violations emphasize the need for sound design and ethical guidance. Inclusive design and ethical considerations are vital for advancing scientific knowledge while preserving human dignity. Informed consent is essential for ethical research and human rights, particularly for vulnerable populations, but it poses challenges in vaccine trials. These principles must be adapted to local conditions and cultural sensitivities.

## 8 Advantages, limitations and development stages of *Helicobacter pylori* vaccines

*H. pylori* vaccines are vital for therapy and prevention, enhancing mucosal immunity and mixed immune responses. The main types are toxin-based and OMP vaccines, with OMP vaccines being safer and more effective because of their lower antigen and adjuvant requirements, potentially reducing antibiotic resistance in aging populations in developing countries. However, viable OMP candidates are limited. Prophylactic vaccination can eradicate bacteria, but challenges exist in identifying suitable antigens and developing effective delivery systems. Toxin vaccines also improve safety but face selection challenges. Innovations such as virus-like particle and DNA vaccines offer new research avenues, although significant hurdles remain before their clinical use. Vaccine development is crucial for preventing *H. pylori* infections and establishing herd immunity. Various vaccine types, including whole-cell, subunit, and epitope vaccines, have been developed, but their efficacy is uncertain. Researchers are also exploring DNA and live vector vaccines, which may activate antibodies or T cells for protective immunity, although the mechanisms are not well understood. Current vaccines do not meet ideal criteria, such as inducing systemic Th1-biased immune responses and stimulating mucosal immunity in gastric tissue. Future efforts should prioritize optimal protection with minimal side effects.

## 9 Future directions

The development of *H. pylori* vaccination strategies requires further preclinical testing in suitable animal models before human trials. A reliable method for assessing vaccine efficacy would aid progress. Research has shown that mice immunized with combinations of five adjuvant *H. pylori* proteins achieve better bacterial clearance. Prime-boost vaccination strategies involving various *H. pylori* antigens have shown significant protective effects in murine models. However, an effective *H. pylori* vaccine has not yet been developed because of limited knowledge of antigens and immune responses. It remains uncertain whether vaccines alone can eradicate *H. pylori* or if antibiotics are needed. The link between *H. pylori* infection and upper gastrointestinal disorders has renewed interest in vaccine development, which could help prevent chronic gastritis, peptic ulcers, gastric cancer, and MALT lymphoma.

## 10 Conclusion

*H. pylori* is implicated in a range of gastric disorders, including peptic ulcers, stomach cancer, lymphoma, and gastritis. The pathogenicity of this bacterium is modulated by several virulence factors, including *cagA*, *vacA*, *ure*, *HP-NAP*, catalase, and OMPs. A comprehensive understanding of these factors is essential for the effective treatment and management of associated conditions. The prevalence of OMPs varies geographically, and all *H. pylori* strains possess these proteins, rendering them potential targets for vaccine development. Current clinical trials are exploring recombinant vaccines that incorporate various antigens, including the *cagA*, *vacA*, *ure*, *babA*, *sabA*, *oipA*, and porin proteins. Recent advancements in antigen detection have opened new avenues for vaccine development, emphasizing innovative delivery methods and adjuvants. Nevertheless, the majority of research remains in preliminary stages. Continued efforts are critical to the development of a safe and effective vaccine against *H. pylori*, which necessitates the identification of immune-suppressing mechanisms, the selection of effective antigens and adjuvants, and the enhancement of public awareness, particularly in developing countries.
